# Objective and Comprehensive Evaluation of Bisulfite Short Read Mapping Tools

**DOI:** 10.1155/2014/472045

**Published:** 2014-04-15

**Authors:** Hong Tran, Jacob Porter, Ming-an Sun, Hehuang Xie, Liqing Zhang

**Affiliations:** ^1^Department of Computer Science, Virginia Tech, Blacksburg, VA 24061, USA; ^2^Virginia Bioinformatics Institute, Virginia Tech, Blacksburg, VA 24061, USA

## Abstract

*Background*. Large-scale bisulfite treatment and short reads sequencing technology allow comprehensive estimation of methylation states of Cs in the genomes of different tissues, cell types, and developmental stages. Accurate characterization of DNA methylation is essential for understanding genotype phenotype association, gene and environment interaction, diseases, and cancer. Aligning bisulfite short reads to a reference genome has been a challenging task. We compared five bisulfite short read mapping tools, BSMAP, Bismark, BS-Seeker, BiSS, and BRAT-BW, representing two classes of mapping algorithms (hash table and suffix/prefix tries). We examined their mapping efficiency (i.e., the percentage of reads that can be mapped to the genomes), usability, running time, and effects of changing default parameter settings using both real and simulated reads. We also investigated how preprocessing data might affect mapping efficiency. * Conclusion*. Among the five programs compared, in terms of mapping efficiency, Bismark performs the best on the real data, followed by BiSS, BSMAP, and finally BRAT-BW and BS-Seeker with very similar performance. If CPU time is not a constraint, Bismark is a good choice of program for mapping bisulfite treated short reads. Data quality impacts a great deal mapping efficiency. Although increasing the number of mismatches allowed can increase mapping efficiency, it not only significantly slows down the program, but also runs the risk of having increased false positives. Therefore, users should carefully set the related parameters depending on the quality of their sequencing data.

## 1. Introduction

DNA methylation is the addition of a methyl group (CH_3_) at the 5th carbon position of the cytosine ring. Most cytosine methylation occurs in the sequence context of 5′CG3′ (also called CpG dinucleotide) in mammalian DNA but some in CpH dinucleotides (where H = C, T, or A). The human genome is not methylated uniformly, and some small regions called CpG islands are usually unmethylated and GC rich. DNA methylation is responsible for regulation of gene expression, silencing of genes on the inactive X chromosome, imprinted genes, and parasitic DNAs [1]. DNA methylation is also a major contributor to the generation of disease-causing germ-line mutations and somatic mutations that cause cancer [2]. Therefore, accurate genome-wide determination of DNA methylation in different cells, tissues, and developmental stages is crucial for identification of causes for phenotype differences and diseases and cancer.

Large-scale characterization of DNA methylation has been made possible by bisulfite conversion of genomic DNA combined with next generation sequencing. After bisulfite treatment of DNAs, unmethylated Cs are converted to Ts and subsequent mapping of the short reads to a reference genome allows inference of methylated versus unmethylated Cs. Thus, inference on DNA methylation is highly dependable on the mapping of bisulfite treated short reads to a reference genome. Similar to regular next generation sequencing analysis, the great challenge is to be able to map thousands of millions of reads in reasonable time and with high mapping efficiency (i.e., the percentage of reads that are mapped to a reference genome).

Many tools have been developed to tackle this computational challenge such as MAQ [3], Bismark [4], BSMAP [5], PASH [6], RMAP [7], GSNAP [8], Novoalign [9], BFAST [10], BRAT-BW [11], Methylcoder [12], CokusAlignment [13], BS-Seeker [14], BS-Seeker2 [15], Segemehl [16], BiSS [17], BatMeth [18], and the latest one ERNE-bs5 [19]. The majority of these bisulfite sequencing mappers first conduct some sequence conversions (e.g., Cs to Ts and Gs to As) either on the reads or the reference genomes, or both and then use existing regular aligners such as Bowtie [20], Bowtie2 [21], BLAT [22], SOAP [23], and BWA [24] to map short reads to a reference genome. Fonseca et al. [25] classified the tools according to their indexing techniques and supported features such as mismatches, splicing, indels, gapped alignment, and minimum and maximum of read lengths. Chatterjee et al. [26] compared Bismark, BSMAP, and RMAPBS in terms of uniquely mapped reads percentages, multiple mapping percentages, CPU running time, and reads mapped per second. They also pointed out that trimming the data before aligning could improve mapping efficiency. However, the study did not examine how setting different parameters might impact program performance.

In this paper, we present how modifying default parameters in each program might change the results (i.e., mapping efficiency and CPU time) and the sensitivity of each program to the characteristics of data. Though we examined many software packages, we mainly focused on two mappers: BSMAP and Bismark since they are representatives of two different index algorithms, namely, Burrows-Wheeler Transform in Bismark and hash table in BSMAP. In general, genome indexing based tools performed better than read indexing tools and read indexing does not provide any significant speedup [27]; therefore, we did not include RMAP in our analysis. We also show that trimming data improves mapping efficiency. The paper is organized as follows: first, we briefly describe the bisulfite sequence mapping problem and mapping techniques used by the tools. Then we describe the datasets used in the study and criteria used to evaluate the performance of the tools. Finally we show results on evaluating the tools using both real and simulated data.

## 2. Overview of the Computational Problem, Algorithms, and Tools

### 2.1. Computational Challenges of Mapping Bisulfite Short Reads

Over the decades, bisulfite sequencing has remained the gold standard for DNA methylation analysis. After bisulfite treatment, unmethylated Cs are converted to thymines (T), whereas methylated Cs are unchanged. Several factors make bisulfite short reads more complicated to map than regular reads. Firstly, up to four strands are analyzed from one genomic region. There are two scenarios after PCR amplification. In the first case, if the sequencing library is generated in a directional manner, the strand that the reads are amplified from is known a priori. However, if nondirectional, the Watson and Crick strands of bisulfite treated sequences are no longer complementary to each other due to the conversion, and there are four different strands after PCR amplification: BSW (bisulfite Watson), BSWR (reverse complement of BSW), BSC (bisulfite Crick), and BSCR (reverse complement of BSC), all amplified and sequenced at roughly the same frequency [13]. The search space is, therefore, significantly increased relative to the original reference sequence [5]. Secondly, sequence complexity is reduced as all unmethylated Cs are changed into Ts. In the mammalian genome, because C methylation occurs almost exclusively at CpG dinucleotide, the majority of Cs in BSW and BSC strands will be converted to Ts. Therefore, most reads from the two strands will be C-poor. However, PCR amplification will complement all Gs with Cs in BSWR and BSCR strands, so reads from these two strands are typically G-poor and have a normal C content. As a result, we expect the overall C content of bisulfite reads to be reduced by approximately 50% after the two processes (converting Cs to Ts in bisulfite treatment and transcribing Gs to Cs in PCR amplification) [5]. Lastly, C to T mapping is asymmetric. The T in the bisulfite reads could be mapped to either C or T in the reference genome but not vice versa. This complicates the mapping process.

### 2.2. Algorithms and Tools for Bisulfite Short Reads Mapping

**Figure 1:**
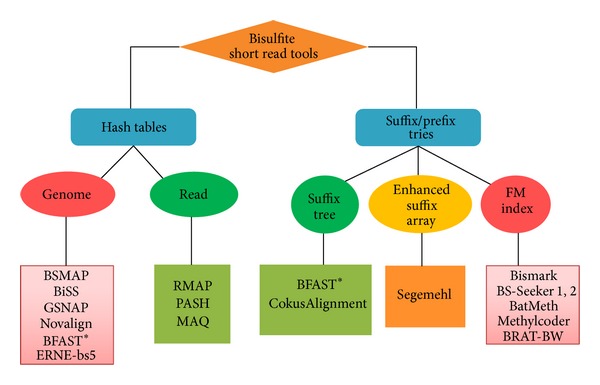
Bisulfite mapping tools classification. The tools can be divided into two groups based on indexing strategies: hash tables or suffix/prefix tries. Each of the groups is classified further into subgroups where some example programs are shown. *BFAST uses multiple index strategies: both hashing and suffix tree.

For most of the existing programs, alignment process is to build auxiliary data structures called indices for the reference genome, the reads or both. The indices are then used to find matching genomic positions for each read. There are many available methods to build the indices [28]. The two most popular techniques are hash tables and suffix/prefix tries [27] reviewed below together with some representative programs (Figure 1). A comprehensive comparison of detailed functionalities of the programs is shown in Table 1.

Indexing using hash tables can be divided into three strategies: hashing the genome, hashing the reads, or a combination of both. All hash table algorithms essentially follow the seed-and-extend technique. The algorithm keeps the positions of each k-mer fragment of the read/genome in a hash table using k-mer as the key and searches the sequence databases for k-mer matches (called seeds) [28]. After this, seeds can be joined without gaps and refined by local sequence alignment. Tools using this indexing technique include BSMAP (genome hashing) [5], GSNAP (genome hashing) [8], Novalign (genome hashing) [9], BFAST (genome hashing/suffix array) [29], RMAP (read hashing) [7], BiSS (genome hashing) [17], PASH (read hashing) [6], MAQ (read hashing) [3], and ERNE-bs5 (genome hashing) [19].

In particular, BSMAP is implemented based on SOAP (Short Oligonucleotide Alignment Program) [23]. BSMAP indexes the reference genome for all possible k-mers using hash tables. BSMAP masks Ts in bisulfite reads as Cs (i.e., reverse bisulfite conversion) only at C position in the original reference and keeps other Ts in the bisulfite reads unchanged. Then BSMAP maps the masked BS read directly to the reference genome. By combining bitwise masking and hash table seeding in its algorithm, BSMAP offers fast and good performance [5].

BiSS (Bisulfite Sequence Scorer) is based on Smith-Waterman local alignment with a customized alignment scoring function [17]. BiSS uses NextGenMap [30] to align bisulfite reads to a reference genome. NextGenMap involves three steps. In the first step, NextGenMap indexes the reference genome in a hash table. The next step is to identify the genomic region match. NextGenMap only considers regions where the number of k-mer matches exceeds a certain threshold as a match. Unlike other methods, NextGenMap adaptively chooses the threshold, meaning each read has different threshold rather than one threshold for all reads [30].

Indexing algorithm based on suffix/prefix tries essentially converts the inexact string matching to exact matching problem. The algorithm involves two steps: identifying exact matches and building inexact alignments supported by exact matches. Several representations for searching exact matches in suffix/prefix tries are suffix tree, enhanced suffix array, and FM-index [28]. Therefore, indexing using suffix/prefix tries can be classified into three subgroups: indexing using suffix tree, indexing using enhanced suffix array, and indexing using FM-index based on Burrows-Wheeler Transform. Tools falling into this category include Bismark (FM index), BS-Seeker (and BS-Seeker2, FM index), BatMeth (FM index), Segemehl (enhanced suffix array), Methylcoder (FM index), Cokus Alignment (suffix tree), and BRAT-BW (FM index).

In particular, in Bismark, bisulfite reads are transformed into a C to T and G to A version (equivalent to a C to T conversion on the reverse strand). Then each of them is aligned to equivalently preconverted forms of the reference genome using four parallel instances of Bowtie or Bowtie2 [4]. Bowtie starts by building an FM index for the reference genome and uses the modified FM index [31] to find the matching location. Bowtie2 are designed to support reads longer than 50 bps. The two versions of Bowtie performed quite differently [27]. This read mapping enables Bismark to uniquely determine the strand origin of a bisulfite read.

BS-Seeker is very much similar to Bismark. The only difference is that BS-Seeker only works well for single-end reads, whereas Bismark can work with both single-end and paired-end reads. Also BS-Seeker can explicitly account for tags generated by certain library construction protocols [14]. BS-Seeker records only unique alignments, defined as those that have no other hits with the same or fewer mismatches in the 3-letter alignment [14].

BRAT-BW is an evolution of BRAT [32]. Two FM indices are built on the positive strand of the reference genome: in the first, Cs are converted to Ts, and, in the second, Gs are converted to As. Original reads with C to T conversion are mapped to the first index and reverse complement reads with all Gs changed to As being mapped to the second index. BRAT-BW uses a multiseed approach similar to Bowtie2 [32].

## 3. Methods

### 3.1. Datasets

We evaluated the tools on three types of data, human blood data (GSM791828), human and mouse brain data (GSE47966), and simulated mouse short read data. First, human blood data, including ten datasets (ID: SRR342552, SRR342553, SRR342554, SRR342555, SRR342556, SRR342557, SRR342558, SRR342559, SRR342560, and SRR342561) were downloaded from NCBI's short reads archive [33]. The DNA short read sequences are nondirectional. Each file in SRA format contains about 23 million single-end whole genome shot gun bisulfite sequence reads from human hematopoietic stem/progenitor cells (HSPCs). The BS-Seq reads are conventional base call qualities that are Sanger/Illumina 1.9 encoded Phred values (Phred33) and trimmed to 76 bps. Second, human and mouse brain data, including ten datasets from human brain [33] and eight datasets from mouse brain [33] were downloaded from NCBI's gene expression omnibus [34]. The DNA bisulfite short read sequences are directional. Each file contains around 100 million single-end whole genome shot gun bisulfite sequence reads from human and mouse frontal cortex in SRA format. The BS-Seq reads are conventional base call qualities that are Illumina HiSeq 2000 encoded Phred values (Phred64) and trimmed to 101 bps. Third, simulated bisulfite short reads data were generated from the mouse and human reference genome (versions mm10 and hg19, resp.) using Sherman simulator [35]. Parameters such as sequencing error, bisulfite conversion rate for cytosines in CG-context, and CH-context in Sherman, are determined based on literature for the mouse data [36] and cytosine methylation reports from Bismark for the human data. Reads with different read lengths were generated to mimic the real mouse and human data. In Particular, for examining the effect of sequencing error on mapping efficiency, 24 datasets were generated from the mouse reference genome by varying the sequencing error from 0 to 4.75% (the error rate is a mean error rate per bp). Each dataset contained 1 million short reads with length of 101 bps and CG conversion rate of 10% (10% of all CG-cytosines will be converted into thymines) and CH conversion rate of 98.5% (98.5% of all CH-cytosines will be converted into thymines). For examining the effect of read length on mapping efficiency, 28 datasets were generated by varying the read length from 40 to 160 bps with sequencing error of 0.16%, CG conversion rate of 10%, and CH conversion rate of 98.5% for the mouse data and with sequencing error of 0.16%, CG and CH conversion rate of 19.73% and 98.9%, respectively for the human data. Both human and mouse reference genomes (hg19 and mm10) were downloaded from Ensembl [37].

### 3.2. Important Parameters in Mapping Tools

Programs often have different default settings for the same parameters that can influence their performance. For example, BiSS sets the default mismatch to be 35% of the read, whereas Bismark sets the equivalent parameter to zero. It is therefore important and fair to compare them on a common ground. Several important parameters that can greatly influence program performance include (1) number of mismatches allowed in the seed (e.g., Bismark); (2) number of mismatches allowed in the read (e.g., BSMAP, BS-Seeker, BiSS, and BRAT-BW); (3) directionality of data library (directional or nondirectional); (4) phred quality score (i.e., whether data have Phred score of 33 or 64). In this study, we examined the effect of these parameters on the performance of the programs and how altering them can influence the final mapping results.

### 3.3. Evaluation Criteria

The performance of the tools is evaluated mainly by two aspects: the mapping efficiency (i.e., percentage of uniquely mapped reads) and the CPU time. Uniquely mapped reads are reads that are mapped to only one location. Computationally speaking, most reads have multiple matches and from those matches, alignment scores are determined. An alignment is unique when it has much higher score than all other possible alignments, often determined by some statistics or cutoffs. The greater the difference between the best alignment score and the second-best alignment score, the more unique the alignment is, and the higher its mapping quality should be [38]. Mapping quality is a nonnegative integer **Q** = −10log⁡⁡10**p**, where **p** is an estimate of the probability that the alignment does not correspond to the read's true point of origin. Mapping quality is sometimes abbreviated MAPQ. (10log⁡⁡10 Pr {mapping position is wrong}).

### 3.4. Data Preprocessing

The original data were processed so reads have better quality scores and consequently can be mapped to reference genomes. Perl programming language was used to trim the tail of a read with residues quality score less than or equal to 2. After removing the tail, if the read length is shorter than 30, the read is also discarded. We use both trimmed and raw data in the analysis for the purpose of comparison of how mapping efficiency can be improved by preprocessing the data.

## 4. Results and Discussion

### 4.1. Performance Comparison of the Programs

Five bisulfite reads mapping tools, BSMAP, Bismark, BS-Seeker, BiSS, and BRAT-BW, were chosen to cover different algorithms discussed in the algorithm overview section (also refer to Table 1). BatMeth, Segmenhl, and ERNE-bs5 were not included as BatMeth failed at last step of the reads alignment, Segmenhl consumed too much computer memory (1 TB) and could not be finished in reasonable time, and ERNE-bs5 produced inaccurate results on small test datasets.

**Figure 2:**
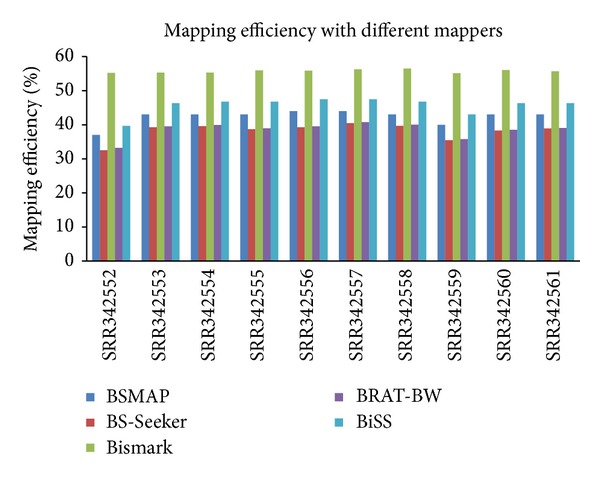
Mapping efficiency on ten human blood datasets for BSMAP, Bismark, BS-Seeker, BRAT-BW, and BiSS with zero mismatches allowed between reads and the reference genome.

The performance is evaluated by considering two factors: mapping efficiency and CPU running time. Mapping efficiency is determined by the number of uniquely mapped reads divided by the total number of reads. We set the number of mismatches to zero for all the programs and compare mapping efficiency and CPU running time of these programs on ten human blood datasets. Among the five programs, in terms of mapping efficiency (Figure 2), Bismark performs the best, achieving the highest mapping efficiency (average around 56% across the ten human blood samples), followed by BiSS (average around 46%) and BSMAP (average around 42%), and finally BRAT-BW (average around 39%) and BS-Seeker (average around 38%) with similar mapping efficiency across samples.

**Figure 3:**
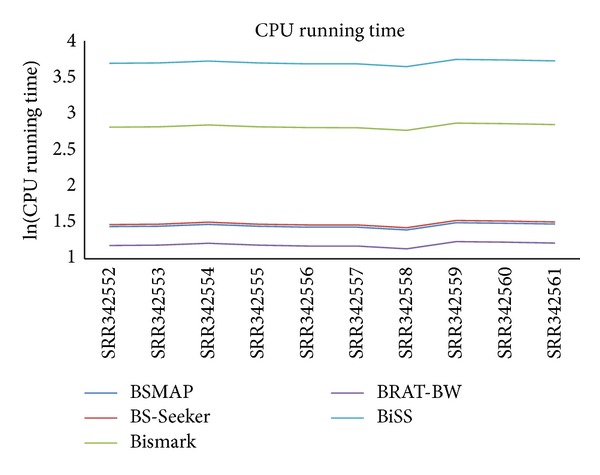
CPU running time (on a log scale) on human blood data for BSMAP, Bismark, BS-Seeker, BRAT-BW, and BiSS with zero mismatches allowed between reads and the reference genome.

However, for CPU running time, the trend is almost the opposite (Figure 3), with BRAT-BW taking the shortest time (average 16 minutes across samples), followed by BSMAP (average 29 minutes) and BS-Seeker (average 31 minutes). Both BiSS (average 84 hours) and Bismark (average 11 hours) took much longer time than the other three programs, suggesting existence of the tradeoff between mapping efficiency and running time. The observation that BiSS ran the slowest might be because BiSS uses Smith-Waterman local sequence alignment algorithm to align reads to potential genomic locations [17]. Interestingly, although both Bismark (written in Perl) and BS-Seeker (written in Python) use Bowtie (or Bowtie2) for short reads mapping, Bismark ran much slower than BS-Seeker but had much higher mapping efficiency. We then used BSMAP and Bismark to map human fetal brain and mouse brain short reads data (refer to Figure 5). Consistent with the results for human blood data, Bismark has higher mapping efficiency but longer CPU running time than BSMAP. The mapping percentages are very similar across samples (Figure 6). However, mapping efficiency for the human and mouse brain data is higher than those for human blood data, consistent with the original research studies [39], suggesting that mapping efficiency is highly dependent upon the specific experiments producing the data.

Even though tools have similar mapping efficiency, reads that are actually mapped (i.e., mapped reads content) might differ among different programs. To examine how much difference the tools have in mapped reads content, we compared uniquely mapped reads from Bismark and BSMAP. On average, for human blood data, uniquely mapped reads shared by both Bismark and BSMAP account for approximately 97% of the total mapped reads by BSMAP and only 69% by Bismark. The numbers change little with different samples. Therefore, most of the mapped reads identified by BSMAP are also identified by Bismark. The difference in mapped reads content between Bismark and BSMAP can be caused by several factors. First, the two use different string matching strategies. Bismark uses Burrows Wheeler transform and FM-indexes for searching and BSMAP hashes the reference genome for searching. In particular, Bismark uses aligner Bowtie2, whereas BSMAP uses aligner SOAP (older version of SOAP2) to map bisulfite short reads. As a result, difference in mapping algorithms can contribute to difference in mapped read content. According to Hatem et al. [27], Bowtie maintained the best throughput with higher mapping percentages, which could be why Bismark maps more reads than BSMAP. Second, determining whether a read is uniquely mapped is rather arbitrary and program specific [40]. Depending on how each program defines “uniquely mapped” computationally, uniquely mapped read content can vary as a result. We also examined whether combining multiple tools to analyze bisulfite short reads could improve the overall mapping efficiency. We used BSMAP and BS-Seeker to align the unmapped reads from Bismark to see how much further BSMAP and BS-Seeker can improve the overall mapping efficiency.  Table 2 shows that using BSMAP to align the unmapped reads from Bismark improves the overall mapping efficiency slightly better than using BS-Seeker (BSMAP: around 4% improvement; BS-Seeker: only 1%). The lesser improvement from BS-Seeker might be due to the fact that both Bismark and BS-Seeker use Bowtie to align reads although they may have different criteria in postprocessing the mapped reads. Overall, results across different datasets indicate that Bismark was able to identify the most uniquely mapped reads, and addition of more programs does not significantly improve mapping efficiency.

### 4.2. Effect of Varying Parameters in Different Tools

**Figure 4:**
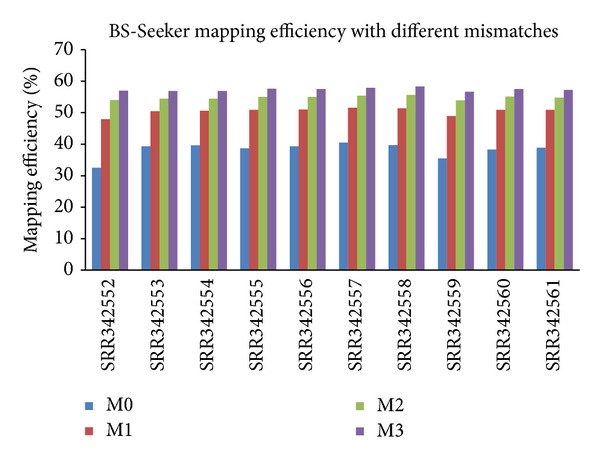
Unique mapping efficiency on ten human blood datasets from BS-Seeker with different numbers of mismatches allowed between reads and the reference genome (0, 1, 2, and 3 mismatches).

We mainly focus on how changing numbers of allowed mismatches between reads and the reference genome affects mapping efficiency. Different programs have parameters that serve this purpose but sometimes have different meanings. For example, BSMAP has the option of setting the number of mismatches allowed in each short read using the parameter **v**. If **v** is between 0 and 1, it is interpreted as the mismatch rate with respect to the read length. Otherwise it is interpreted as the maximum number of mismatches allowed in a read. The default is 0.08. The maximum number of mismatches allowed is 15 per read. BiSS has the option of setting the number of mismatches allowed in each short read using the parameter **i** (minimum identity between a read and a match) ranging from 0 to 1. The default setting is 0.65, meaning 65% of a read and its corresponding match are identical. All reads mapped with an identity lower than this threshold will be reported as unmapped. Our results on changing these parameters show that, in general, the mapping efficiency increases with the number of mismatches. The results are consistent across datasets and for all the programs tested. For brevity, only the results from BS-Seeker were used to illustrate (Figure 4). BS-Seeker has the option of setting the number of mismatches allowed in each short read using the parameter **m**. The default is 2 and the maximum number allowed is 3. Figure 4 shows that with the number of mismatches allowed increasing from 0 to 3, mapping efficiency increases by 43%–60%. Worth noting is that with mapping efficiency increasing, CPU running time also increases significantly. Therefore, in real practice, though it is desirable to have high mapping efficiency, CPU time is another important aspect that users need to consider before running the programs. Sometimes cost of having high mapping efficiency becomes inhibitive as it takes too much running time. For example, when we changed Bismark's allowed mismatches from 0 to 1, the time it takes to finish the program doubles (e.g., increased from 657 to 1581 minutes to run on sample SRR342553). Another important aspect to consider is that increasing the number of mismatches allowed also runs the risk of increased false positives, although in real practice it is difficult to determine whether mapped reads having mismatches to the mapped location are actually false positives or real variants from the reference genome.

### 4.3. Effect of Data Preprocessing

**Figure 5:**
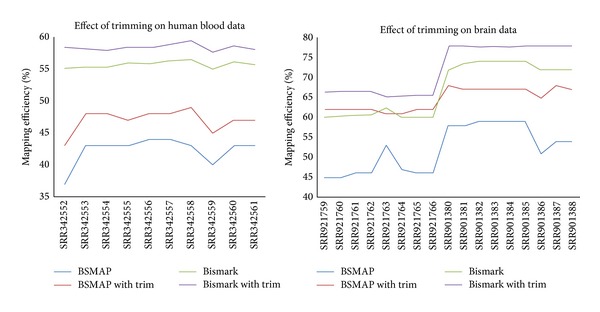
The effect of trimming reads on mapping efficiency on ten human blood, ten human brain, and eight mouse brain datasets for BSMAP and Bismark.

**Figure 6:**
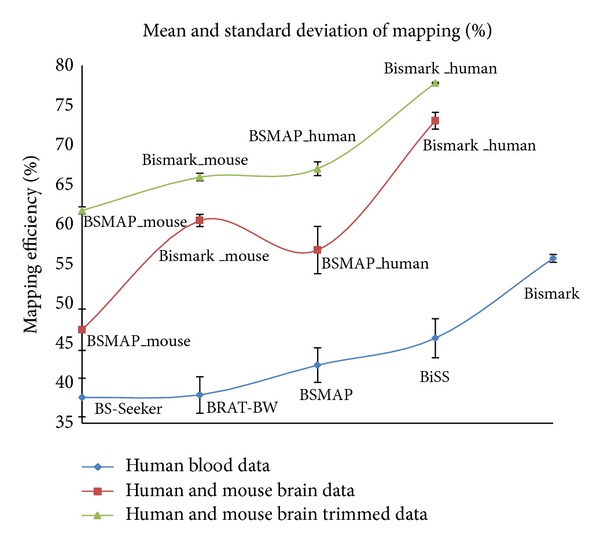
Mean and standard deviations of mapping percentages across ten human blood, ten human brain, and eight mouse brain datasets.

We also preprocessed the reads and used those tools to analyze the trimmed data. Around 2%–4.5% of the blood data and around 1.1%–2.3% were trimmed on the brain data. Figure 5 shows that the mapping efficiency increases by around 5% for BSMAP and around 3% for Bismark on the human blood data and by around 10% for BSMAP and around 6% for Bismark on the human fetal brain and mouse brain data. Therefore, preprocessing reads before mapping is an effective approach to improve mapping efficiency.

### 4.4. Effect of Read Length and Sequencing Error

**Figure 7:**
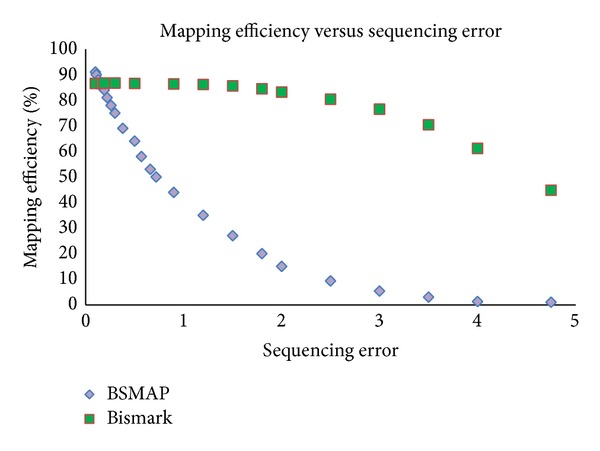
The effect of sequencing error on mapping efficiency for BSMAP and Bismark using simulated data generated from Sherman simulator with varying sequencing error from 0.1 to 4.75% (e.g., sequencing error 0.1% means 1 error in every 1000 bases) for read length = 101 bp, CG = 10% (10% of all CG-cytosines will be converted into thymines), and CH = 98.5% (98.5% of all CH-cytosines will be converted into thymines).

We used simulated data to see the effect of sequencing error and read length on mapping efficiency. Sequencing error has been found to be an important factor influencing the performance of short reads mapping tools [3]. Consistent with previous finding, our result shows that for both BSMAP and Bismark, as sequencing error increases, mapping efficiency decreases (Figure 7). Comparatively, BSMAP is more sensitive to sequencing error than Bismark as the BSMAP's mapping efficiency decays exponentially with the increase of sequencing error, while Bismark's only gradually.

**Figure 8:**
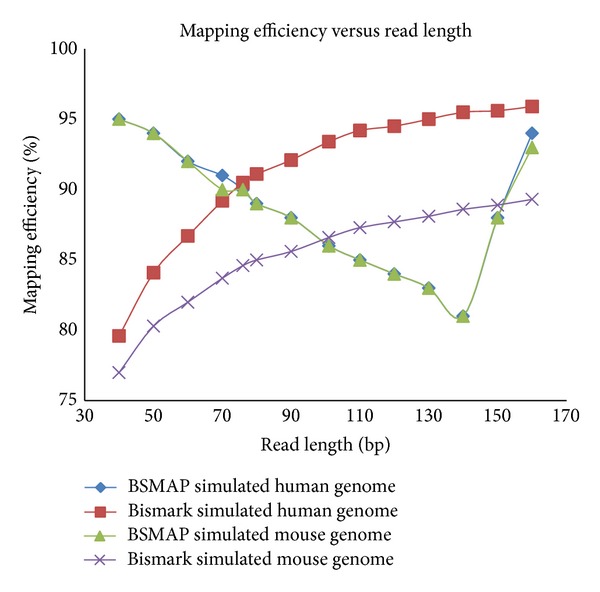
The effect of read length on mapping efficiency for BSMAP and Bismark using simulated data generated from Sherman simulator with different read lengths (from 40 to 160 bps) for sequencing error e = 0.16, CG = 10%, and CH = 98.5% for mouse and e = 0.16, CG = 19.73%, and CH = 98.9% for human.

Read length is another important factor in short reads mapping.  Figure 8 shows opposite patterns for BSMAP and Bismark. For BSMAP, as read length increases from 40 to 140 bps, mapping efficiency decreases but with read length above 140 bps, an increase in read length results in an increase in mapping efficiency. On the other hand, unique mapping efficiency from BISMARK increases as read lengths increase consistently. It is unclear what contributes to the pattern exhibited by BSMAP.

## 5. Conclusion

Many bisulfite short read mapping tools are available and choosing the best one among them is a difficult task. In our experiments, even though Bismark produced the highest unique mapping efficiency on real data, its CPU running time was not the shortest. BRAT-BW ran the fastest on real data but with lower mapping efficiency. Also, preprocessing data before mapping can increase mapping efficiency regardless of what tools are used. Changing parameters in the program can affect the mapping results. Overall, as number of mismatches increases, mapping efficiency increases. Short reads length and sequencing error can affect the results. Bismark is more sensitive to read lengths. The longer the read length, the higher the mapping efficiency for Bismark, whereas there is no clear pattern for BSMAP. BSMAP is more sensitive to sequencing error. A small increase in sequencing error can result in significant decrease in mapping efficiency from BSMAP.

## Figures and Tables

**Table 1:** Detailed comparison of different bisulfite short reads mapping tools.

Programs	Year	Algorithmic Technique used	Language	Aligner	Input	Output	Min./Max. read length	Mismatches	Indels	Gaps	Single/Paired-end	Multi-threaded	Nondirectional
ERNE-bs5	2012	Hash genome indexing uses a 5-letter (Cm, Cu) for storing methylation information and uses a weighted context-aware Hamming distance to identify a T coming from an unmethylated C.	C++	None	gz/bz2/fastq/fasta	BAM/ SAM	up to 600 bp	1 every 15 bp (-errors arg)	Yes	Yes	both	Yes	No

BatMeth	2012	FM index integrates mismatch counting, list filtering and mismatch stage filtering and fast mapping onto two indexes.	Perl/C++	None	fasta	NA	NA	up to 5 (-*n*) in a read	No	No	Yes	Yes	Yes

BiSS	2012	Reference genome hashing, local Smith-Waterman alignment	Perl	None	fasta/fastq/gz/SAM/BAM	SAM/BAM/Next GenMap	up to 4096 bp	(-*i* from 0 to 1) in a read Default *i* = 65%	Yes	Yes	Yes	Yes	No

Bismark	2011	FM-Index enumerates all possible T to C conversion	Perl	Bowtie/Bowtie2	fasta/fastq	BAM/SAM	Bowtie: up to 1000 bp Bowtie 2: unlimited	0 or 1 in a seed (-*N*)	Yes	Yes	both	Yes	Yes

BS-Seeker2	2013	FM-Index enumerates all possible T to C conversion	Python	Bowtie2/Bowtie/SOAP/RMAP	fasta, fastq, qseq, pure sequence	BAM/SAM/BS-Seeker	50–500 bp	up to 4 per read (-*m*)	Yes	Yes	Single	No	Yes

BS-Seeker	2010	FM-Index, enumerates all possible T to C conversion, converts the genome to 3 letters, and uses Bowtie to align reads	Python	Bowtie	fasta, fastq, qseq, pure sequence	BAM/SAM/BS_Seeker	50–250 bp	up to 3 per read (-*m*)	Yes	No	Single	No	Yes

BSMAP	2009	hashing of reference genome and bitwise masking tries all possible T to C combinations for reads	Python	SOAP	fasta/fastq/ SAM	SAM/txt	up to 144 bp	up to 15 in a read (-*v*)		up to 3 bp	both	Yes	Yes

RMAP	2008	Wildcard matching for mapping Ts, incorporates the use of quality scores directly into the mapping process	C++		fastq/fasta	BED	unlimited	up to 10 in a read (-*m*)	No	No	both	No	No

BRAT-BW	2012	Converts a TA reference and CG reference; two FM indices are built on the positive strand of the reference genome	C++		Text file with input file names in fastq, sequence only	txt	32 bp-unlimited	unlimited	No	No	both	Yes	Yes

MAQ	2008	Builds multiple hash tables to index the reads, scans the reference genome against the hash tables to find hits	Perl/C/C++		fastq	maq	Up to 63 bp	up to 3 per read	Yes, -*n* = 2	No	both	No	No

PASH	2010	Implements *k*-mer level alignment using multipositional hash tables	C		fastq	Txt/SAM	NA	Yes	Yes	No	Single	No	No

Novo-align	2010	Hashing genome	C/C++		fastq	SAM/BAM	up to 8 per read, 16 for paired end reads	Yes	Yes	up to 7 bp on single end reads	Both	No	Yes

Methyl-coder	2011	FM-Index, all Cs converted to Ts	C/C++/Python	GSNAP/bowtie	fastq/fasta	BAM/SAM	Bowtie: up to 1000 bp	Yes	No	Yes	both	No	No

GSNAP	2005	*q*-mer hashing of reference genome	C/Perl		gzip/fastq, fasta/bzip2	SAM/GSNAP	14–250 bp	Yes	Yes	Yes	both	yes	No

BFAST	2009	Uses multiple indexing strategies: hashing and suffix array of the reference genome	C		fastq/bz2/gzip	SAM	NA	Yes	Yes	Yes	both	Yes	Yes

Segemehl	2008	Enhanced suffix arrays to find exact and inexact matches. Align to read using Myers bitvector algorithm	C/C++		fasta	SAM	unlimited	Yes	(-*A* ^∗1^)	Yes	both	Yes	No

*BFAST does not have a direct option for bisulfite mapping, users have to convert Cs to Ts in both a reference genome and reads and then align converted reads to the converted reference genome.

*Parenthesis in mismatches column indicates parameter for mismatches in a program.

^∗1^A min percentages of matches per read.

**Table 2:** Improvement in mapping efficiency after using BSMAP and BS-Seeker to map unmapped reads from Bismark on human blood data.

File name	Total number of reads	Unmapped reads in BISMARK	Overall improvement using BSMAP	Overall improvement using BS-Seeker
SRR342552	23,472,574	10512269	3.72%	0.90%
SRR342553	23,749,583	10610307	4.24%	1.03%
SRR342554	25,232,053	11277407	4.29%	1.07%
SRR342555	23,750,428	10452979	4.23%	1.01%
SRR342556	23,140,352	10204603	4.28%	1.06%
SRR342557	23,089,492	10093756	4.33%	1.05%
SRR342558	21,205,564	9215604	4.26%	1.04%
SRR342560	26,174,056	11491673	4.17%	1.01%
SRR342561	25,457,341	11271400	4.16%	1.02%
